# Outcomes of Threaded Intramedullary Headless Nail Fixation for Metacarpal Fractures

**DOI:** 10.7759/cureus.48618

**Published:** 2023-11-10

**Authors:** Alec Giron, Cameron T Cox, Erin Choi, Gracie Baum, Desirae McKee, Brendan J MacKay

**Affiliations:** 1 Orthopaedic Surgery, Texas Tech University Health Sciences Center School of Medicine, Lubbock, USA; 2 Orthopaedic Surgery, Texas Tech University Health Sciences Center, Lubbock, USA

**Keywords:** threaded intramedullary nail, intramedullary nail, intramedullary fixation, metacarpal fracture fixation, metacarpal fracture

## Abstract

Metacarpal fractures are common and may require operative treatment; however, there is no gold standard fixation technique. K-wires are used for simplicity and flexibility but require immobilization and can lead to complications. Dorsal plating provides greater stability than K-wires but is subject to its own limitations. Intramedullary threadless nails have reduced time to union and improved range of motion (ROM) but may not provide the stability of plating. Intramedullary screws allow rapid return to activity and increased stability; however, compression forces may shorten metacarpals, producing non-anatomic reduction.

The ExsoMed INnate^TM ^threaded intramedullary nail (ExsoMed, Aliso Viejo, CA, USA) was developed with tall threads, a diameter fitting the metacarpal canal cortical purchase, stability, and no compression to prevent fracture displacement and shortening. We designed a prospective study to evaluate INnate^TM^ nail fixation in metacarpal fractures. Visual analog scale (VAS) pain score, total active motion (TAM), radiographic union, and return to normal activity were recorded at each postoperative visit. Descriptive statistics were performed for endpoints.

Fifty-eight patients were included with a median age of 38.7 years. The INnate^TM^ nail was used in 80 fractured metacarpals. The 4th and 5th metacarpals were most frequently involved. The most common mechanisms of injury were blunt trauma and motor vehicle collisions. Approximately 31% of patients had multiple, concurrent fractures (16 patients required two nails; two required three nails). The mean follow-up was 9.9 weeks. The mean time to radiographic union was 9.1 weeks. The final visual analog scale (VAS) pain score was 1.1. The mean time to return to work and/or normal activities was 9.7 weeks, and the mean percentage of normal activity resumed was 89.1%.

## Introduction

Metacarpal fractures are common, particularly among young males (age 10-40), comprising 30% of all hand fractures and 18% of all below-elbow fractures in the United States [1,2]. Despite their frequent occurrence, treatment modalities for metacarpal fractures are varied and continue to evolve. While many metacarpal fractures can be treated nonoperatively, there is a growing tendency to choose operative treatment as it may prevent deformity and mobility deficits in these often young and active patients [3]. Fixation techniques vary by patient characteristics and type of fracture [4].

Historically, percutaneous K-wires have been used due to their simplicity [5-9]. Unfortunately, these can tether the surrounding tissue, especially the sagittal bands, limiting the range of motion (ROM) in the early stages (up to 3-4 months) of healing and rehabilitation [4]. Pin tract infections also remain a risk of K-wire fixation [4]. Dorsal metacarpal plating has been used as a straightforward alternative to K-wires pinning. Plating offers greater stability but has been associated with complication rates as high as 35% including tendon adhesion, persistent stiffness, hardware failure, and infection [6-9].

Given the limitations of K-wires and dorsal plating, Gonzalez et al. developed a technique using pre-bent, flexible intramedullary nails for internal stabilization of metacarpal fractures [10]. This technique was improved by Orbay and Touhami by adding a locking pin to the proximal end of the nail [5]. Outcomes of this fixation technique compare favorably to K-wire fixation with an average time to union of 6.3-8.0 weeks and a 16-day earlier return to work [3,4,11,12].

More recently, intramedullary compression screws have been used to hold reduction and prevent rotational instability. These headless screws are buried within the metacarpal and do not need to be removed. Intramedullary compression screws allow for early mobilization (5 days or earlier) while providing fixation strength comparable to traditional techniques [13]. Early studies evaluating this technique have shown improved functional outcomes [13-22].

The INnate^TM^ threaded intramedullary nail (ExsoMed, Aliso Viejo, CA, USA) was developed with increased thread height and diameter comparable to metacarpals to create cortical purchase and stability. Increased thread pitch was added to control rotation and speed up insertion and extraction. Non-compressive thread design was added to prevent shortening and fracture displacement during device insertion. Threaded intermedullary nails have previously been shown to have improved rotational stability when compared to K-wire fixation, with a higher load to failure [23]. Currently, very few studies exist that compare INnate^TM^ intramedullary nails to other more commonly used techniques. Given the paucity of data on this novel product and the lack of consensus gold standard fixation methods, we designed a prospective study evaluating the utility of the INnate^TM^ intramedullary threaded nail in metacarpal fracture fixation.

Indications for use of the INnate^TM^ nail include, but are not limited to fixation of intra-articular and extra-articular fractures and non-of small bones and bone fragments in the scaphoid, carpals, metacarpals, tarsals, and metatarsals. Contraindications for the use of INnate^TM^ Nail fixation include active or latent infection or sepsis, insufficient quantity or quality of bone and/or soft tissue to support fixation, and suspicion of material sensitivity.

## Materials and methods

Approval was obtained from the Texas Tech University Health Sciences Center (TTUHSC) Lubbock/Odessa Institutional Review Board (approval number: L20-034), and appropriate informed consent as well as any necessary HIPAA consent was obtained. This study was performed in accordance with the ethical standards laid down in the 1964 Declaration of Helsinki and its later amendments. Fifty-eight patients (80 fractured metacarpals) were treated with INnate^TM^ intramedullary nail fixation and were prospectively included in our study (Table 1).

**Table 1:** Distribution of metacarpal fractures in the INnateTM intramedullary nail cohort INnate^TM^ threaded intramedullary nail (ExsoMed, Aliso Viejo, CA, USA).

Placement	Number Injured
2^nd^ Metacarpal	9 (11.3%)
3^rd^ Metacarpal	10 (12.5%)
4^th^ Metacarpal	30 (37.5%)
5^th^ Metacarpal	31 (38.8%)
Total:	80 (100%)
Injuries involving multiple metacarpals	Number of Patients
4^th^ & 5^th^ Metacarpals	12
3^rd^ & 4^th^ Metacarpals	2
2^nd^ & 3^rd^ Metacarpals	2
2^nd^, 3^rd^, & 4^th^ Metacarpals	1
3^rd^, 4^th^, & 5^th^ Metacarpals	1

**Figure 1:**
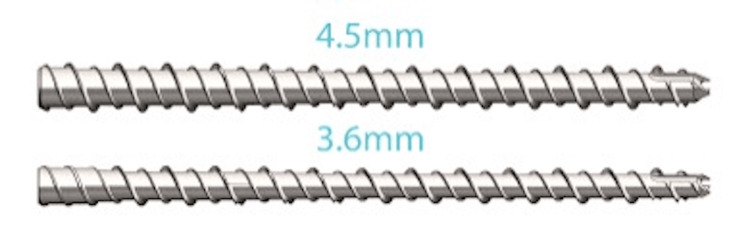
INnateTM intramedullary threaded nails. INnateTM threaded intramedullary nail (ExsoMed, Aliso Viejo, CA, USA).

**Figure 2:**
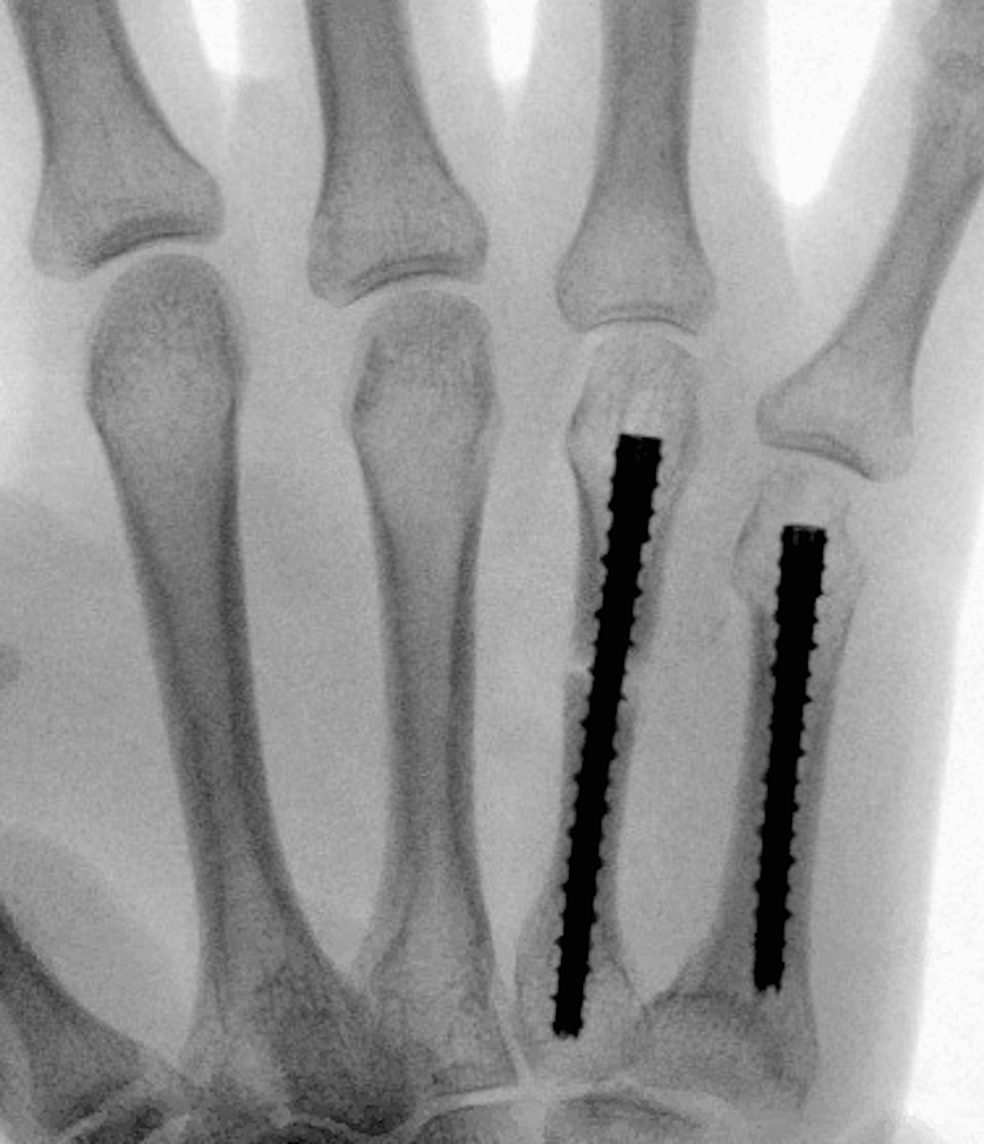
Intraoperative fluoroscopic image The image shows INnateTM intramedullary threaded nail placement in the fourth and fifth metacarpals.

The surgical repair involved the use of either a 3.6 or 4.5mm INnate^TM^ nail, with nail lengths varying from 25 to 65 mm (Figure 1). The surgical technique involved longitudinal incision of the skin, extensor tendon, and capsule. In accordance with the INnate^TM^ surgical technique guide, a K-wire was used to measure the length of each metacarpal and provide preliminary fixation. Appropriate INnate^TM^ nails were then selected, and placed under fluoroscopic guidance (Figure 2). Fluoroscopic evaluation and intraoperative physical exam including gross inspection and wrist tenodesis were performed to evaluate for correct metacarpal rotation.

Data was collected at routine follow-up visits. Outcome measures included visual analog scale (VAS) pain scores, total active motion (TAM), time to radiographic union, and return to normal activities. Descriptive statistics were performed to evaluate endpoints.

## Results

A total of 58 patients (41 males, 17 females) were included. Median age was 38.7 (range: 14-88). 80 fractured metacarpals were treated with ExsoMed INnate^TM^ intramedullary nailing. The 4th (37.5%) and 5th (38.8%) metacarpals were the most commonly injured. The most common mechanism of injury was blunt trauma, followed by motor vehicle collision (Table 1). Approximately 31% of patients had multiple, concurrent metacarpal fractures (16 patients required two nails; two patients required three nails) (Table 1). Two patients had concurrent fractures that required fixation across the carpometacarpal (CMC) joint, one with a proximal 4th metacarpal fracture and one with a displaced intra-articular 5th metacarpal base fracture, in which both nails were advanced into the hamate for stability.

The mean follow-up interval was 9.9 weeks (range: 0.9-68.7 weeks). The final VAS pain score was 1.1 (range: 0-7). 60% of patients had 100% TAM, 16% had 90-99% TAM, 14% had 75-89% TAM, 4% had 50-74% TAM, and 6% had 25-49% TAM (Table 2). The mean time to radiographic union was 9.1 weeks (range: 5-23 weeks). The mean time to return to work and/or normal activities was 9.7 weeks (range: 1-25.6 weeks), and the mean percentage of normal activity resumed was 89.1% (range: 25-100%).

**Table 2:** Outcomes of INnateTM intramedullary threaded nail fixation for metacarpal fractures at mean follow-up of 9.9 weeks NnateTM threaded intramedullary nail (ExsoMed, Aliso Viejo, CA, USA).

Variable	Mean ± St. Dev.
(Number of fractures)	(Range)
Radiographic union (weeks)	9.1 ± 3.8
(n = 45)	(5-23)
VAS Pain (points)	1.1 ± 2.1
(n = 68)	(0-7)
Mean % Total Active Motion (TAM)	88.7 ± 19.6 %
(n = 50)	(25-100)
100% TAM	60%
90-99% TAM	16%
75-89% TAM	14%
50-74% TAM	4%
25-49% TAM	6%
% Normal Activity Resumed	93.3 ± 14.3 %
(n = 41)	(25-100)
Return to Work/Daily Activities	9.7 ± 5.3 weeks
(n = 36)	(1-25.6)

There were no complications or revision procedures related to fracture fixation in our cohort. Thorough evaluation for infection, bone quantity and quality, and relevant soft tissue structures may mitigate the risk of complications.

## Discussion

Metacarpal fractures are classified as open, closed, intra- or extra-articular and described as oblique, transverse, spiral, or comminuted. The degree of angulation is an important consideration of the treatment algorithm as each metacarpal can tolerate a different amount of angulation before operative care is recommended. The ring and middle fingers tolerate the least amount of angulation, which can be as low as 10° [24]. There is some physiologic compensation for angulation via hyperextension of the metacarpophalangeal (MCP) joint, however, as angulation increases, there is an inverse relation to the force generated at the proximal interphalangeal joint. This decrease in force leads to an extensor lag, also known as pseudoclawing [24]. Another consideration is the shortening of the fractured metacarpal which can create problems for the extensor mechanism.

Intra-articular fractures and fractures resulting in malrotation require special consideration. In the case of malrotation, there can be prominent flexor dysfunction leading to scissoring of the fingers when making a fist. Each degree of rotation at the metacarpal can have as much as 1.5cm overlap among fingers when making a closed fist [24]. Operative treatment has been recommended for intra-articular fractures in which 25% of the articular surface is involved or a step-off of 2 mm or more is present [24].

Current fixation techniques can be limited in their utility to provide satisfactory outcomes, particularly in cases where metacarpal shortening is of concern. Percutaneous pinning with K-wires has commonly been used for internal fixation of metacarpal fractures due to ease of availability and simplicity (Table 3) [5-9]. However, K-wires are associated with several complications including infection, loss of reduction, symptomatic non-union, and pin loosening [4]. They are limited in their utility to treat spiral or comminuted fractures [25]. Decreased construct rigidity often necessitates immobilization with a protective splint or cast, which can delay the range of motion and rehabilitation compared to other fixation techniques [24].

**Table 3:** Intramedullary nail or K-wire fixation outcomes for metacarpal fractures

Reference	Number of Fractures	VAS Pain (Range)	Total Active Motion (Range)	Grip Strength (Range)	Radiographic Union (Range)	Return to Daily Activities (Range)
Orbay and Touhami, 2006 [5] - 1.1 or 1.6-mm flexible non-locking intramedullary nail	34	1.4 (1-2.5)	239 (230-270)	89%	5.9 weeks	n/a
Orbay and Touhami, 2006 [5] - 1.1 or 1.6-mm flexible locking intramedullary nail	76	1.3 (1-2.75)	244 (225-275)	92%	5.6 weeks	n/a
Ozer et al., 2008 [6] - Intramedullary nailing (Hand Innovations, Inc.)	38	n/a	237 (150-270)	n/a	5.4 weeks	
Facca et al., 2010 [7] - 1.6 or 2-mm intramedullary K-wire	20	0.9	97.7%	92.9%	n/a	8.1 weeks
Fujitani et al., 2012 [8] - 2 x 1.2-mm intramedullary K-wires with bouquet technique	15	n/a	94%	67%	2.3 months	2.3 months
Dreyfuss et al., 2019 [9] - 1 or 2 x 1.2-mm intramedullary K-wires	39	n/a	92.7%	83% (40-110)	7.1 weeks (4-41)	

As previously mentioned, plate fixation is more stable than percutaneous pinning with K-wires, and can address a variety of fracture patterns including spiral, long oblique, short oblique, and transverse fractures (Table 4) [6-9]. Additionally, dorsal plating has resulted in improved ROM when compared to K-wires [26]. One study showed mini-plates have a slightly shorter union time (11.80 versus 12.95 weeks) and non-statistically significant lower rates of complication (including infection, implant loosening, loss of reduction, and stiffness) when compared to K-wire fixation [27]. Yet, complications have been reported as high as 36% following plate fixation of metacarpal and phalangeal fractures, leaving up to 34% of patients with extensor lag or stiffness and 19% with MCP or proximal interphalangeal (PIP) contracture [28]. A more recent study noted stiffness as the most frequent complication, occurring in 40% of patients [29]. This is likely due to the open nature of plate fixation and plate to soft tissue interaction which can lead to increased scar tissue and adhesions [1].

**Table 4:** Plate fixation outcomes for metacarpal fractures

Reference	Number of Fractures	VAS Pain (Range)	Total Active Motion (Range)	Grip Strength (Range)	Radiographic Union (Range)	Return to Daily Activities (Range)
Ozer et al., 2008 [6] - Plate-screw fixation (Synthes)	14	n/a	228^o^ (150-270)	n/a	5.2 weeks (4-7)	
Facca et al., 2010 [7] - Locking plates (Aptus Hand®, Médartis^TM^)	18	0.94	58.7%	88.4%	n/a	7.4 weeks
Fujitani et al., 2012 [8] - Low profile plates	15	n/a	96%	86%	2.0 months	n/a
Dreyfuss et al., 2019 [9] - Locking plates (Variable Angle Locking Hand System, Depuy Synthes & Hand Fracture System, Acumed)	35	n/a	98.0%	93% (42-125)	8.4 weeks (5.3-15)	

Intramedullary nail fixation provides increased stability compared to K-wire fixation (Table 4) [4,6-9]. When compared to K-wires, intramedullary nails provided earlier recovery of ROM, earlier return to work, and decreased complication rates [4]. One retrospective study of 66 metacarpal fractures fixated with intramedullary nails reported an average union time of seven weeks, with nine (14%) delayed unions (>three months) and no non-unions [3]. There were three cases (5%) of nail migration, two cases of skin impingement (3%), two cases (3%) of rotational deformities, and one infection (2%) [3].

Compression screws have significant advantages over percutaneous K-wires and open reduction and internal fixation (ORIF) techniques (Table 5) [14-22]. One notable improvement is the ability to provide increased stability in simple fractures [13,30]. Multiple studies have demonstrated that early ROM and adequate time to radiographic union can be achieved without immobilization (Table 5) [13-21,30]. A study of 91 patients who were treated with headless intramedullary screws reported that all patients achieved full active MCP motion at most recent follow-up, with an average grip strength of 104.1% of the contralateral hand. Active motion was initiated 5 days postoperatively, and mean time to radiographic union was 6 weeks [30]. Despite promising outcomes data, some studies have questioned the stability of these screws in some fracture patterns. A cadaveric study compared the biomechanical stability of compression screws to other fixation techniques and found significantly lower load at failure and lower number of cycles to failure during a cyclic loading protocol [31]. Another study found that in unstable metacarpal fractures, dorsal plating was found to provide superior stability when compared to compression screws [32].

**Table 5:** Intramedullary screw fixation outcomes for metacarpal fractures.

Reference	Number of Fractures	VAS Pain (Range)	Total Active Motion (TAM)	Grip Strength (Range)	Radiographic Union (Range)	Return to Daily Activities (Range)
Boulton et al., 2010 [14] - 3.0-mm cannulated headless compression screw; length, 45 mm (Synthes)	1	n/a	80^o^ Flexion, Complete extension	n/a	By 12 weeks	By 6 weeks
Doarn et al., 2014 [15] - 3.0-mm cannulated headless screw; length, 40 mm (Synthes); long thread for neck, short thread for shaft	9	0	100%	98%	7 weeks (4-12)	6 weeks (4-10)
Ruchelsman et al., 2014 [16] - 2.4-mm or 3.0-mm headless compression screw; length NA (Synthes)	39	n/a	100%	105% (58-230)	By 6 weeks	n/a
del Piñal et al., 2015 [17] - 3.0-mm headless cannulated screw; length, 40 mm or 4.0-mm; length 50 mm for fifth metacarpal (Small Bone Innovations)	48	n/a	249^o^	n/a	n/a	11 weeks (3.5-64.5)
Tobert et al., 2016 [13] - 3.0-mm partially threaded; length, 32–45 mm (Synthes)	18	n/a	All >240^o^	n/a	n/a	n/a
Lee et al., 2017 [18] - 3.0-mm headless compression screw; length to fit in head only (Synthes)	5	n/a	265^o^	n/a	5 weeks (5-7)	n/a
Romo-Rodriguez et al., 2017 [19] - 3.0-mm, 4.0-mm; length, 26–44 mm	10	n/a	90% of patients had 100% TAM	n/a	By 4 weeks	By 4 weeks
Couceiro et al., 2018 [20] - 2.4-mm, 3.0-mm headless compression screw; length n/a	19	1 (0-4)	96%	88.3%	n/a	4 weeks (2-6)
Jann et al., 2018 [21] - 2.2-mm, 3.0-mm; length, ≤40 mm	20	n/a	85% of patients had 100% TAM	93%	4 weeks (4-6)	n/a
Siddiqui et al., 2019 [22] - 2.4-mm or 3.0-mm headless compression screw; length 30-50 mm	35	0	243^o^	88.6%	6.6 weeks	3.6 weeks

Threaded intramedullary nails provide a novel approach to metacarpal fracture fixation, including benefits that are not supported by other techniques. These threaded nails share similar benefits to compression screws such as improved rotational stability compared to K-wire fixation [23]. The outcomes in our cohort are similar to studies using intramedullary nails and intramedullary compression screws [3,30]. The INnate^TM^ nail allowed stable fixation in our cohort without the need for immobilization and resulted in a comparable time to radiographic union. Threaded intramedullary nails pose less concern for compression and resultant shortening when compared to compression screws. This quality may be particularly valuable in the repair of unstable or complex fractures, in which the compression screw may not be optimal for retaining metacarpal length.

Our study was limited by a lack of a control group for direct comparison of threaded intramedullary nails with historical fixation techniques. Additionally, we did not analyze the impact of pre-existing patient factors or injury mechanisms on time to radiographic union, and further studies are needed to clarify the role of these variables in determining recovery trajectory.

## Conclusions

Our cohort included complex, open, and comminuted fractures. There is a paucity of literature assessing compression screw efficacy in complex fractures, as most studies exclude patients with open fractures or intra-articular fractures. Our data suggests that the INnate^TM^ threaded intramedullary nail can provide equivalent surgical outcomes across a broader spectrum of fractures and/or concomitant injury patterns, thus improving and potentially simplifying metacarpal fracture treatment algorithms.
